# Rasch analysis of the Multiple Sclerosis Impact Scale (MSIS-29)

**DOI:** 10.1186/1477-7525-7-58

**Published:** 2009-06-22

**Authors:** Melina Ramp, Fary Khan, Rose Anne Misajon, Julie F Pallant

**Affiliations:** 1 Department of General Practice, University of Melbourne, 200 Berkeley Street, Carlton 3053, Victoria, Australia; 2 Department of Rehabilitation Medicine, University of Melbourne, Royal Melbourne Hospital, Melbourne, Australia; 3 School of Political and Social Inquiry, Monash University, Melbourne, Australia; 4 School of Rural Health, University of Melbourne, 49 Graham Street, Shepparton 3630, Victoria, Australia

## Abstract

**Background:**

Multiple Sclerosis (MS) is a degenerative neurological disease that causes impairments, including spasticity, pain, fatigue, and bladder dysfunction, which negatively impact on quality of life. The Multiple Sclerosis Impact Scale (MSIS-29) is a disease-specific health-related quality of life (HRQoL) instrument, developed using the patient's perspective on disease impact. It consists of two subscales assessing the physical (MSIS-29-PHYS) and psychological (MSIS-29-PSYCH) impact of MS. Although previous studies have found support for the psychometric properties of the MSIS-29 using traditional methods of scale evaluation, the scale has not been subjected to a detailed Rasch analysis. Therefore, the objective of this study was to use Rasch analysis to assess the internal validity of the scale, and its response format, item fit, targeting, internal consistency and dimensionality.

**Methods:**

Ninety-two persons with definite MS residing in the community were recruited from a tertiary hospital database. Patients completed the MSIS-29 as part of a larger study. Rasch analysis was undertaken to assess the psychometric properties of the MSIS-29.

**Results:**

Rasch analysis showed overall support for the psychometric properties of the two MSIS-29 subscales, however it was necessary to reduce the response format of the MSIS-29-PHYS to a 3-point response scale. Both subscales were unidimensional, had good internal consistency, and were free from item bias for sex and age. Dimensionality testing indicated it was not appropriate to combine the two subscales to form a total MSIS score.

**Conclusion:**

In this first study to use Rasch analysis to fully assess the psychometric properties of the MSIS-29 support was found for the two subscales but not for the use of the total scale. Further use of Rasch analysis on the MSIS-29 in larger and broader samples is recommended to confirm these findings.

## Background

Multiple Sclerosis (MS) is a chronic and degenerative neurological disease affecting an estimated 2.5 million people worldwide [1]. MS causes a number of impairments such as motor weakness, spasticity and incoordination, pain, fatigue, blurred vision, sensory dysasthesias, depression, anxiety, and bladder dysfunction. These have a significant impact on a person's daily living activities (function), participation and quality of life (QoL) [2-7]. QoL and changes following treatment in persons with MS are difficult to measure despite recent advances in MS treatments (medications), and support for adjuvant and supportive interventions such as rehabilitation [8,9].

A number of measurement scales, both generic and disease specific, have been used to assess the quality of life and functioning of patients with MS. These include the Kurtzke Expanded Disability Status Scale (EDSS) [10], Multiple Sclerosis Quality of Life (MSQOL-54) [11], the Multiple Sclerosis Quality of Life Inventory (MSQLI) [12], the Functional Assessment of Multiple Sclerosis (FAMS) [13], the Multiple Sclerosis Functional Composite (MSFC) [14], the UK Neurological Disability Scale (UKNDS) [15], the Health-Related Quality of Life Questionnaire for Multiple Sclerosis (HRQOL-MS) [16] and the Medical Outcomes 36-item Short-Form Health Survey (SF-36) [17].

One scale that is increasingly being used in both research and clinical settings is the Multiple Sclerosis Impact Scale (MSIS-29) [18]. This scale was developed as a disease-specific measure of HRQoL using the patient's perspective on disease impact in MS. It was designed to be a scientifically rigorous, clinically useful, disease specific instrument to enhance existing measures used in treatment trials, developed *from *and completed *by *patients with MS, ensuring measurement of HRQoL outcomes relevant to those patients that are sometimes overlooked by clinicians [2,19]. The scale's authors engaged standard psychometric techniques of classical test theory (CTT) to construct the MSIS-29, on a United Kingdom community sample with MS [18]. Using factor analysis, they identified two dimensions that mirrored previous findings of patients' views of health status, which they labelled *physical impact *and *psychological impact *[18]. The physical impact dimension (MSIS-29-PHYS: consisting of 20 items) and the psychological impact dimension (MSIS-29-PSYCH: consisting of nine items) measure related, but distinct, constructs (correlatingr r = 0.62), yielding two separate scale scores, with higher scores indicating higher impact level [18]. Although a total impact score can be calculated, the authors do not recommend its use in clinical trials or epidemiological research, as combining the subscales conceals possible diverging effects of treatment on the two health dimensions [18].

A number of studies have re-examined the psychometric properties of the MSIS-29 using CTT methods [19-25]. Each study has shown overall support for the original findings on the MSIS-29 physical and psychological scale's data quality, scaling assumptions, acceptability, reliability, external validity and responsiveness to change in various settings and in a number of MS populations in the UK [19,21,24], Ireland [20,23], the Netherlands [22,26] and, more recently, Iran [25]. None of these subsequent studies, however, re-examined the internal factor structure of the MSIS-29 or its subscales.

As with all new scales, the MSIS-29 requires further validation in a variety of settings and samples, and utilizing different methodologies [18,19,21]. In particular the scale authors suggest that the MSIS-29 should be subjected to validation with newer psychometric methods such as Rasch analysis [18,19,21]. Rasch analysis, which was originally developed by Danish mathematician Georg Rasch [27], is increasingly being used in the development and evaluation of clinical tools for the health and medical sciences [28]. Rasch analysis provides the opportunity to evaluate many aspects of a scale's functioning, including a detailed assessment of response format, item content, response bias, dimensionality and appropriate targeting of a scale [29-31]. It also allows the transformation of ordinal level scale scores into equal interval measurement, which is particularly important when measuring change or responsiveness to treatment [28,29]. Only scales that fit the Rasch model fulfil the requirements of objective conjoint measurement required for mathematical manipulation of the scores.

To date, no study has made full use of the features available in Rasch analysis to investigate the internal validity of the MSIS-29. Two previous studies used Rasch analysis to assess specific issues that arose in the course of their research [23,26]. In addition to a study conducted in the Netherlands [26], an Irish study showed support for the MSIS-29 physical subscale's divergent validity using Rasch calibrated interval level scale scores [23].

With the increasing use of the MSIS-29 in research in many parts of the world further analysis of the scale's psychometric properties needs to be undertaken. The aim of this study was to use Rasch analysis to further test the internal validity of the MSIS-29 subscales (MSIS-29-PHYS, MSIS-29-PSYCH) and total scale (MSIS-29-TOTAL) in terms of response format, item fit, model fit, item bias, targeting, internal consistency and dimensionality in an Australian sample.

## Methods

### Participants and setting

This study involves secondary analysis of data collected for a larger study conducted in Melbourne, Australia evaluating the efficacy of rehabilitation in multiple sclerosis patients [8]. Ethics approval for this study was granted by the Royal Melbourne Hospital (RMH) Human Research and Ethics Committees. A community sample of persons aged between 18–65 years with 'definite' MS (McDonald criteria [32]) were recruited using the RMH MS Database [8]. Of the 101 eligible patients who consented to the broader study, 92 patients [66 (72%) women; 26 (28%) men] were included in the current cross-sectional study, having completed the MSIS-29 at baseline. These participants ranged in age from 29 to 65 years with a mean age of 50.45 years (SD = 8.96 years). Years since diagnosis ranged from 1 to 43 years (M = 10.56, SD = 7.40) with 30% (N = 26) classified as Relapsing/remitting, 58% (N = 53) as Secondary Progressive and the remainder (11, 12%) as Primary Progressive. Twenty percent (N = 18) recorded Expanded Disability Status Scale (EDSS) [10] scores between 0 and 3, 58% (N = 53) scored between 3.5 and 6.0 and 23% (N = 21) scored 6.5 and above.

### Procedure & materials

As part of the larger study [8] participants were asked to complete a questionnaire booklet which included the MSIS-29 [18]. The MSIS-29 is a 29 item scale consisting of two subscales, a 20-item scale measuring physical impact (MSIS-29-PHYS) and a 9-item scale measuring psychological impact (MSIS-29-PSYCH) [18]. All items have a polytomous response format (range 1–5), with higher scores indicating higher impact level. A total score for each subscale can be derived by summing items and transforming them into a score out of 100. The authors suggest a total scale score can also be calculated (MSIS-29-TOTAL)[18], however they advise caution against its use in clinical trials and epidemiological studies. The MSIS-29 has been shown to have good internal consistency, with Cronbach's alpha values for each of the subscales of between 0.87 and 0.96 [18,19,21,23]. A number of studies have shown overall support for the convergent and discriminant construct validity of the MSIS-29-PHYS and MSIS-29-PSYCH [19,20,22].

### Statistical analyses

Separate Rasch analyses were conducted to assess the internal validity of the MSIS-29-PHYS, MSIS-29-PSYCH and MSIS-29-TOTAL using RUMM2020 software [33]. Each set of items was assessed for threshold ordering, overall model fit, item fit, person fit, reliability, differential item functioning, targeting and dimensionality. The procedures adopted were consistent with the published guidelines of Tennant and colleagues [34,31].

Before conducting Rasch analyses a decision needs to be made as to whether the rating scale model or the partial credit model should be used to analyse the scales [29]. The partial credit model, unlike the rating scale model, does not require assumptions to be met regarding item distribution [34]. It allows items to have different numbers of response categories [29], and does not assume the distance between response thresholds is uniform for all items [34]. RUMM2020 provides the likelihood ratio test to identify the form of Rasch model that is most appropriate for a given set of data. A significant likelihood ratio test indicates that the distance between response thresholds is inconsistent, and therefore, a partial credit model is preferred [34].

For a scale to have good fit to the Rasch model it is expected that patients with high levels of the measured attribute (e.g. physical or psychological impact of MS), will consistently endorse high scoring response options across all items and patients with low levels will endorse low scoring responses. All items are expected to have ordered response thresholds, with the term *threshold *signifying the point between adjacent response categories where either response is equally probable. Disordered thresholds occur when respondents inconsistently endorse response categories. This can be the result of ambiguous response labelling or too many response options. The presence of disordered thresholds can be detected from the threshold map provided by RUMM2020, and the extent of the disordering determined by inspection of the category probability curves for each item [34]. Disordered thresholds can be resolved, where considered necessary, by collapsing adjacent response categories.

Model fit was assessed using three summary statistics. Good overall model fit was indicated by a non-significant item-trait interaction chi-square probability value, indicating the hierarchical ordering of items is consistent over all levels of the trait. Item and person fit are indicated by two item-person interaction fit residuals transformed to approximate z-scores, with a mean of zero and standard deviation of one indicating perfect fit to the model. Residuals and chi-square probability values of individual items or persons were inspected, with misfit indicated by fit residual values > ± 2.5 and/or chi-square probability values < 0.05 (using a Bonferonni adjustment to the alpha level for the number of items) [34]. High positive fit residual values indicate misfit to the model and high negative fit residuals indicate redundancy. Internal consistency of the scale was estimated by the Person Separation Index (PSI), which is interpreted in the same way as a Cronbach's alpha coefficient, with values above 0.7 considered acceptable [30,31].

Differential item functioning (DIF) was assessed to examine potential item bias caused by different groups (gender, age groups) in the sample. An analysis of variance is conducted for each item to examine scores over different levels of the person factor, and over different levels of the underlying trait (using a Bonferroni adjusted alpha level) [34]. The current study assessed potential DIF on sex and age group (< 53 years/≥ 53 years).

To assess the ability of the scales to appropriately target the population being measured, person-item threshold distribution maps were inspected. A well targeted scale should include a set of items that span the full range of person estimates.

Possible response dependency among the items was investigated by inspecting the residual correlation matrix [34] for pairs of items with correlations exceeding 0.3. Dimensionality of the scales was assessed using independent t-tests to compare person estimates derived from the two most disparate subsets of scale items [35]. These subsets of items are defined by positive and negative loadings on the first factor extracted using a principal component analysis of residuals. For a scale to be considered unidimensional no more than five percent of cases should show a significant difference between their scores on the two subsets [34]. If more than five percent show a difference a binomial test of proportions is used to calculate the 95% confidence interval around the t-test estimate. Unidimensionality is said to be supported if the value of five percent falls within the 95% confidence intervals [34,36].

The sample size for Rasch analysis varies according to a number of parameters, including the degree of required precision of the person and item estimates, and the targeting of the sample. A well targeted sample is one in which the person distribution closely matches the item distribution when they are both calibrated on the same metric scale. A sample size of 64 cases is considered sufficient to give a stable item calibration within ± 0.5 logit where the sample is well targeted, rising to 144 when the sample is poorly targeted [33,37].

Rasch calibrated person ability estimates for both subscales were imported into SPSS [38] from RUMM2020 and the correlation between the two subscales was assessed using a Spearman's correlation coefficient (rho).

## Results

### Rasch analysis

Likelihood ratio tests for all three scales were significant, supporting the use of a partial credit Rasch model (MSIS-29-PHYS: *p *< 0.001; MSIS-29-PSYCH: *p *= 0.015; MSIS-29-TOTAL: *p *< 0.001).

### MSIS-29 physical impact scale

The threshold map for the 20 items of the MSIS-29-PHYS indicated that over half (11/20) of the items had some degree of threshold disordering. A number of rescoring options were tested, however, a global rescore was found to be the most appropriate solution. All disordered thresholds were resolved by reducing the original 5-point response scale (12345) to a 3-point scale (01112), by merging the three middle response categories (*'a little'*, *'moderately' *and *'quite a bit'*).

The overall fit statistics for the rescored MSIS-29-PHYS showed good model fit (p = 0.363) and high internal consistency (PSI = 0.93) (see Analysis 2, Table 1). The mean fit residual value for items was -0.12 (SD = 1.29) showing good individual item fit to the Rasch model, however, individual person fit statistics showed some misfitting persons, with a mean fit residual of -0.44 (SD = 1.64).

**Table 1:** Summary fit statistics for original and final models for MSIS-29 subscales

**Action**	**Analysis Number**	**Overall** **model fit**	**Item fit Mean** **(SD)**	**Person fit Mean** **(SD)**	**PSI**	**% significant t-tests**
**MSIS-29-PHYS**						
						
Original scale	1	χ^2 ^= 44.04, df = 20, p = .002	.31 (1.46)	-.09 (1.33)	.95	

Rescoring to 3-point	2	χ^2 ^= 21.59, df = 20, p = .36	-.12 (1.29)	-.44 (1.64)	.93	

Removal of 1 case	3	χ^2 ^= 19.34, df = 20, p = .50	-.10 (1.19)	-.44 (1.58)	.93	9.21% (CI:4–14%)
						
**MSIS-29-PSYCH**						
						
Original scale	4	χ^2 ^= 12.57, df = 9, p = .18	.09 (1.28)	-.38 (1.58)	.90	

Removal of 1 case	5	χ^2 ^= 14.79, df = 8, p = .10	.10 (1.31)	-.33 (1.49)	.91	1.69%

PSI = Person Separation Index, SD = standard deviation, df = degrees of freedom, p = probability, CI = confidence interval

One case had a positive fit residual above 2.5 (3.22). After this person's data was removed person fit and overall model fit improved (see Analysis 3 – Table 1). In the final solution one item (item 20: *needing to go to the toilet urgently*) recorded a positive fit residual above 2.5 (3.72). Given the Bonferroni adjusted p-value for the item was non-significant and overall model fit was achieved it was decided to retain this item (see Table 2).

**Table 2:** Individual item fit statistics for the 20-item MSIS-29-PHYS

**Item**	**MSIS Item name**	**Location**	**SE**	**Fit Resid**	**Chi Sq**	**Prob**
1	Do physically demanding tasks	-2.239	0.254	0.382	0.632	0.427

2	Grip things tightly (e.g. turning on taps)	0.219	0.201	0.453	1.509	0.219

3	Carry things	-0.788	0.235	0.361	0.138	0.710

4	Problems with your balance	-0.481	0.279	0.747	0.259	0.611

5	Difficulties moving about indoors	1.682	0.256	0.294	0.156	0.693

6	Being clumsy	0.061	0.278	-1.097	2.381	0.123

7	Stiffness	0.009	0.233	-0.200	0.424	0.515

8	Heavy arms and/or legs	-0.258	0.218	-0.758	0.089	0.765

9	Tremor of your arms or legs	0.696	0.213	1.356	1.091	0.296

10	Spasms in your limbs	0.723	0.222	0.574	0.375	0.540

11	Your body not doing what you want it to do	-0.577	0.204	-0.669	0.115	0.734

12	Having to depend on others to do things for you	-0.102	0.219	-1.681	2.116	0.146

13	Limitations in your social and leisure activities at home	0.515	0.234	-0.938	4.469	0.035

14	Being stuck at home more than you would like to be	0.022	0.196	-0.721	0.148	0.701

15	Difficulties using your hands in everyday tasks	0.669	0.219	-0.268	0.384	0.536

16	Having to cut down the amount of time you spent on work or other daily activities	-0.096	0.215	-0.702	0.033	0.855

17	Problems using transport (e.g. car, bus, train, taxi, etc.)	0.639	0.199	-0.916	0.258	0.611

18	Taking longer to do things	-0.200	0.239	-0.884	0.086	0.770

19	Difficulty doing things spontaneously (e.g. going out on the spur of the moment)	-0.075	0.206	-1.117	0.025	0.875

20	Needing to go to the toilet urgently	-0.419	0.196	**3.715**	4.649	0.031

Values showing significant misfit bolded.

SE = Standard error, Fit Resid = Fit Residual, Chi-Sq = Chi-Square, Prob = probability

Fit Residual df = 82.6, Chi-square df = 1

**Figure 1:**
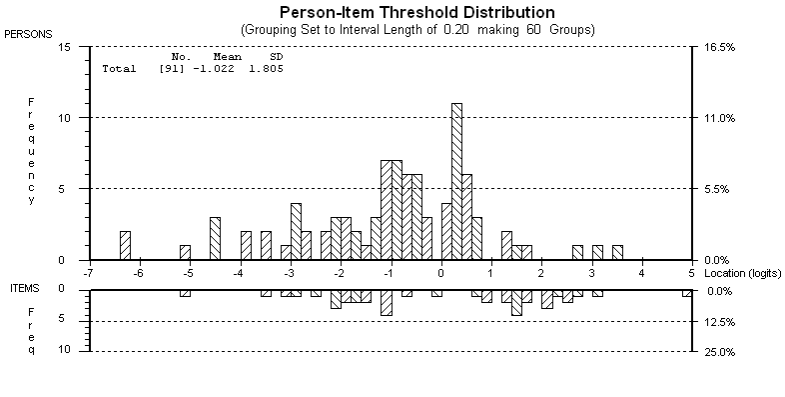
**Targeting map for the 20-item MSIS-29-PHYS after rescoring and removal of one person (N = 91)**.

No differential item functioning was found for sex or age group (under 53 years/53 years and over). The targeting map for the MSIS-29-PHYS shows that the items and thresholds adequately spanned the full range of person scores except for a few persons recording very high or very low scores (see Figure 1). This suggests that the MSIS-29-PHYS is well targeted for the current sample of MS patients.

No response dependency among the items was detected, with all correlations among the items in the residual correlation matrix falling below 0.3. Dimensionality of the rescored MSIS-29-PHYS was assessed using a Principal Component Analysis of the residuals to detect the two most disparate subsets of scale items, suggested by positive and negative loadings on the first component extracted. Results from a series of paired samples t-tests used to compare person estimates on the two most disparate subsets showed support for the unidimensionality of the MSIS-29-PHYS. Although seven of the 76 (9.21%) patients showed significantly different scores on the two subsets, the 95% confidence intervals around this estimate, derived from a binomial distribution, included five percent (CI: 4% to 14%), indicating that the unidimensionality of the scale was supported.

### MSIS-29 psychological impact scale

The 9-item MSIS-29-PSYCH showed good fit to the Rasch model (p = 0.18) with good internal consistency (PSI = .90) (see Analysis 4 – Table 1). The threshold map showed disordered thresholds for three items (items 21, 22 & 26). As the level of disorder revealed in the category probability curves for these items was relatively minor and model fit was satisfactory, the original 5-point response format was retained.

Individual person fit statistics showed some misfitting persons with a mean fit residual value of -0.38 (SD = 1.58). Inspection of individual person estimates showed one person with a fit residual exceeding 2.5 (2.96). After removal of this case the person fit statistics improved, and the PSI increased (PSI = .91) (see Analysis 5 – Table 1). Individual item fit statistics showed one misfitting item (item 22: *problems sleeping*) with a positive fit residual exceeding 2.5 (3.30) [31]. However, given the item had a non-significant Bonferroni adjusted chi-square p-value and that overall model fit was good, the item was retained (see Table 3). No differential item functioning on the MSIS-29-PSYCH was found for sex or age group.

**Table 3:** Individual item fit statistics for the nine-item MSIS-29-PSYCH

**Item**	**MSIS Item name**	**Location**	**SE**	**Fit Resid**	**Chi Sq**	**Prob**
21	Feeling unwell	0.077	0.127	0.434	0.866	0.352

22	Problems sleeping	-0.068	0.120	**3.297**	5.738	0.017

23	Feeling mentally fatigued	-0.346	0.137	-0.342	0.834	0.361

24	Worries related to your MS	-0.348	0.124	-0.110	1.199	0.273

25	Feeling anxious or tense	-0.191	0.134	-0.768	1.299	0.254

26	Feeling irritable, impatient, or short tempered	-0.206	0.141	0.452	0.096	0.757

27	Problems concentrating	-0.059	0.141	-1.217	2.563	0.109

28	Lack of confidence	0.903	0.141	-0.428	2.167	0.141

29	Feeling depressed	0.238	0.136	-0.424	0.028	0.866

Values showing significant misfit bolded.

Fit Residual df = 69, Chi-square df = 1

SE = Standard error, Fit Resid = Fit Residual, Chi-Sq = Chi-Square, Prob = probability

**Figure 2:**
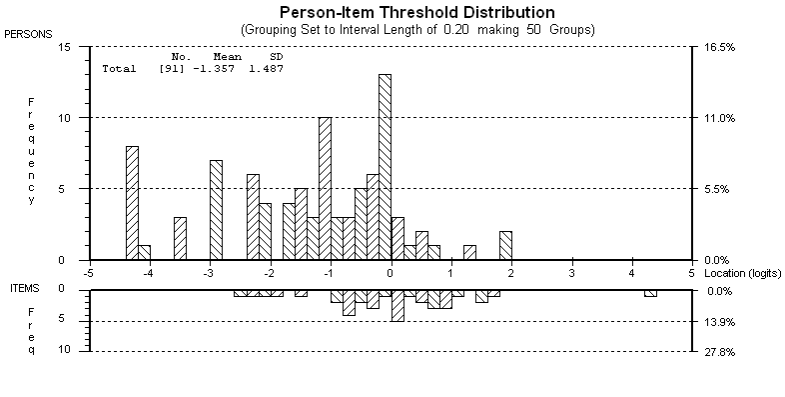
**Targeting map for the nine-item MSIS-29-PSYCH after removal of one person (N = 91)**.

The targeting map for the MSIS-29-PSYCH showed the items and thresholds spanned the range of person scores except for those at the lower impact end (see Figure 2). Independent t-tests comparing person ability estimates on the two most opposing item subsets showed a significant difference in scores for only one of the 59 t-tests (1.69%). These results provide support for the unidimensionality of the MSIS-29-PSYCH.

### MSIS-29 total scale

The MSIS-29-TOTAL was then subjected to Rasch analysis. Item thresholds of all 29 items were checked for disordering. As a large proportion of items (18 of the 29 items) were found to have some level of disordering, a global rescore of items was performed to resolve this problem. Collapsing the original 5-point scale (12345) into a 3-point scale (01112) resolved all disordering.

To establish whether the total scale represents a single underlying construct the dimensionality of the total rescored MSIS-29-TOTAL was examined, prior to generating model fit statistics. Independent t-tests comparing scores from the two most disparate subsets of items failed to find support for the unidimensionality of the total scale with 11 out of 71 t-tests showing a significant difference (15.49%, 95% CI: 10%, 21%). As expected, the items representing the MSIS-29-PHYS and MSIS-29-PSYCH separated into different factors. These results indicate that it is not appropriate to combine the physical and psychological items of the MSIS-29 to produce a total scale score. As unidimensionality is an essential requirement for Rasch analysis no further analyses were conducted on the combined set of items.

Spearman correlation coefficients were calculated from Rasch derived person estimates to assess the intercorrelation between the MSIS-29-PHYS and MSIS-29-PSYCH. There was a relatively strong correlation (rho = 0.62) between these two scales, consistent with Hobart and colleagues' original finding that they measure similar but distinct constructs [18].

## Discussion

Although previous studies using classical theory based techniques have found support for the psychometric properties of the MSIS-29, this is the first study to systematically assess all aspects of the scale using Rasch analysis. Overall support was established for the psychometric properties of the individual scales of the MSIS-29 (MSIS-29-PHYS, MSIS-29-PSYCH) with adequate fit to the Rasch model, no differential item bias, good internal consistency and support for both the targeting and unidimensionality of the scales. A number of issues emerged however in relation to the response format of items, and the fit of some items. The results also suggest that it is not appropriate to combine the two subscales to form a total MSIS score.

Inspection of the item response format for the MSIS-29-PHYS, MSIS-29-PSYCH and MSIS-29-TOTAL revealed issues regarding the ordering of response categories. Substantial disordering of thresholds was identified for many of the MSIS-29-PHYS items, and minor disordering was identified for some of the MSIS-29-PSYCH items. A reduction in number of response categories, from five to three, for the MSIS-29-PHYS items resolved any disordering. No rescoring was undertaken for the MSIS-29-PSYCH items due to the relatively minor degree of disordering.

Problems with the response format in this study may be influenced by the relatively small sample used in this study and will need to be verified in larger and broader samples before specific recommendations can be made. It does however suggest that some modifications to the response format used in this test may be warranted in future studies to reduce potential confusion for respondents. These results suggest that they are unable to reliably distinguish between what is meant by the terms *'a little', 'moderately' and 'quite a bit'*; which could be replaced by a single category (e.g. *moderately*). Despite the reduction in response options from five to three the revised MSIS-29-PHYS scale showed good internal consistency (PSI = .93), supporting this revision.

The rescored MSIS-29-PHYS and MSIS-29-PSYCH scales each revealed a misfitting item (item 20: *needing to go to the toilet urgently*; and item 22: *problems sleeping*, respectively). Given the non-significant Bonferroni adjusted p-values for each item, and that good fit to the Rasch model for each scale was achieved, these items were retained.

The Rasch assumption of unidimensionality was supported for both the rescored MSIS-29-PHYS and MSIS-29-PSYCH. This finding replicated the original structure of the scale reported in earlier studies using factor analytic techniques of CTT [18]. Unidimensionality was not satisfied for the MSIS-29-TOTAL, with results clearly showing that the two scales should not be combined to produce a total score. Consistent with previous research [18,19,22,23], the current study showed that the two MSIS-29 scales measure related, but distinct constructs, as evidenced by an inter-correlation of *rho *= 0.62. The authors of the MSIS-29 were justified in their caution that a total scale score should not be used for clinical trials and epidemiological purposes [18].

Although controversy exists over the appropriateness of reducing human attributes to quantifiable scores [29], measurement of outcomes such as HRQoL can provide a more complete picture of disease burden. This is particularly pertinent for clinical trials of MS due to the disease's heterogeneous presentation, diverse symptoms and unpredictable path, as well as its high prevalence and the lack of a cure. This study provides further evidence for the internal validity of the physical and psychological impact scales of the MSIS-29, supporting their use in clinical and research settings as a measure of HRQoL to augment medical models of disease impact. This study also highlights the contribution that Rasch analysis can make in the evaluation of scales, over and above the classical test theory methods that have dominated the area of scale development in the health and social sciences.

The sample size of 92 MS patients was considered adequate for a Rasch analysis of the MSIS-29 [37], however future studies utilising larger samples should be undertaken using Rasch analysis to confirm the findings of this study. Broader samples, including a wider variety of patients with MS drawn from different settings, should also be utilized. Further evaluation of the response format of the scales should be undertaken to examine the decision made in this study to rescore the response categories of the MSIS-29-PHYS items. Ideally this would include the administration of the original and revised version of the MSIS scoring to the same people to compare their validity. Longitudinal studies should also be undertaken to assess the responsiveness of the MSIS-29 over time, using Rasch calibrated interval-scaled scores. For clinical studies evaluating the efficacy of various treatment and rehabilitation interventions it is important to accurately calculate change scores.

## Conclusion

The MSIS-29 was designed as a disease-specific HRQoL instrument to augment existing measures for MS clinical trials. It was developed and evaluated using techniques based within classical test theory. The current study was the first to undertake a rigorous examination of the MSIS-29 scales' psychometric properties using Rasch analysis. Using an Australian MS sample this study found support for the internal validity, internal consistency reliability, targeting, and unidimensionality of the two MSIS-29 subscales. Modifications to the scoring format was necessary for the MSIS-29-PHYS, however, further validation of this is necessary before clinical implementation would be recommended. The summation of all items to form a total scale was not supported. The further use of Rasch analysis on the MSIS-29 in larger and broader samples is recommended to confirm the findings of the current study.

## Competing interests

The authors declare that they have no competing interests.

## Authors' contributions

MR conducted the data analysis and prepared a draft of the manuscript. FK designed the study and collected the data. RM participated in the preparation and revision of the manuscript. JP supervised the design of the study, data analysis and preparation of the manuscript. All authors contributed to the preparation of the manuscript and read and approved the final manuscript.
